# Supplementary material for: A Global Screen for Assembly State Changes of the Mitotic Proteome by SEC-SWATH-MS
Source: Cell Syst. 2020 Feb 26;10(2):133–155.e6. doi: 10.1016/j.cels.2020.01.001 (PMC7042714; doi:10.1016/j.cels.2020.01.001)

P46934 | NEDD4_HUMAN | NEDD4 KIAA0093 NEDD4−1 PIG53
Monomer MW [kDa]: 149.114 Monomer expected elution fraction: 37

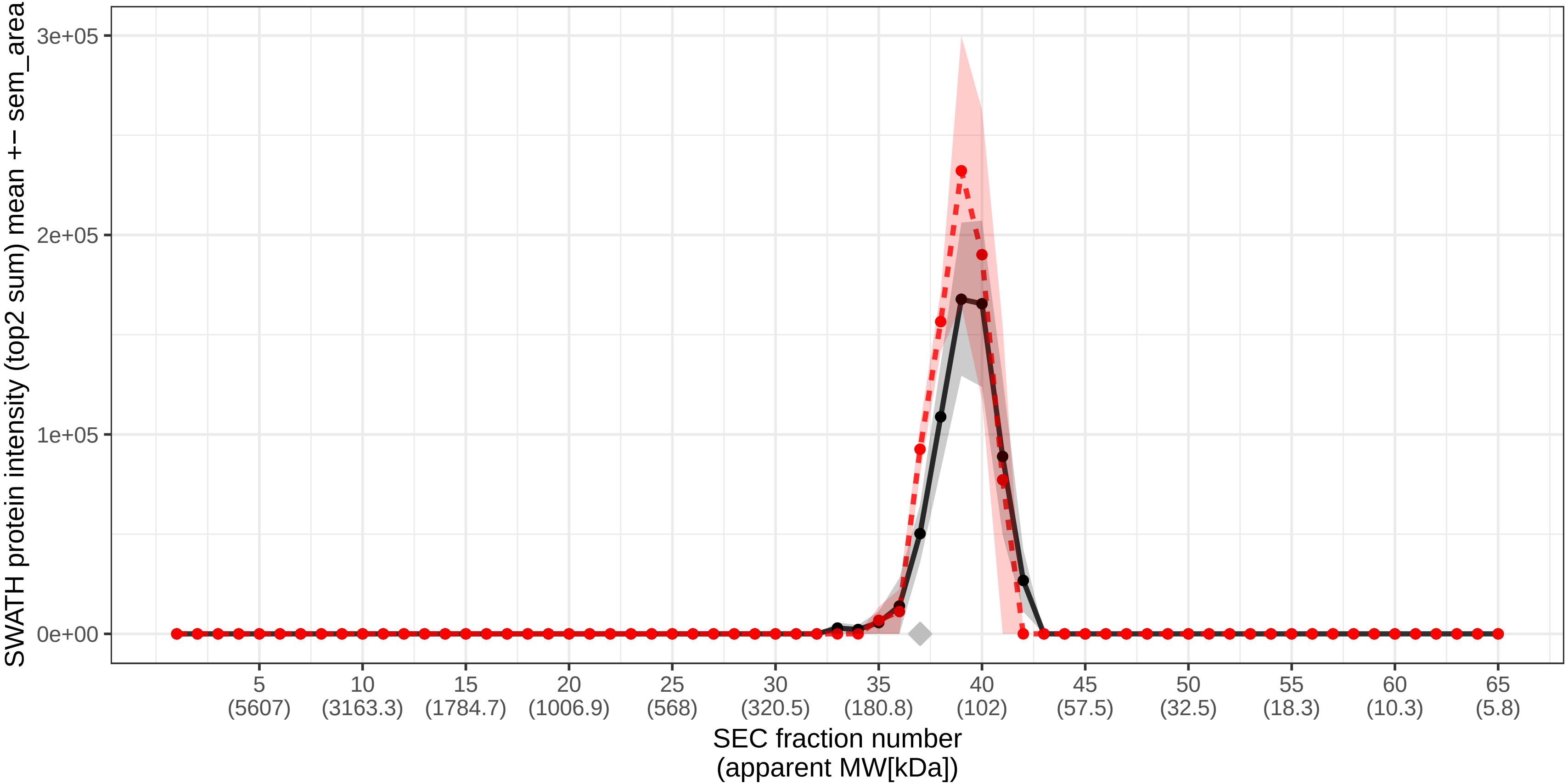

Supplement: Data S1. SEC-SWATH-MS Protein Chromatograms, Related to Figure 1 [file mmc6.zip › SECchrom_P46934_NEDD4_HUMAN_NEDD4_KIAA0093_NEDD4-1_PIG53.pdf]
